# Supplementary material for: Machine Learning Accelerates Crystallization for Structure Determination
Source: Angew Chem Int Ed Engl. 2026 May 4;65(25):e1218503. doi: 10.1002/anie.1218503 (PMC13266958; doi:10.1002/anie.1218503)

checkCIF/PLATON report

Structure factors have been supplied for datablock(s) lcz045

THIS REPORT IS FOR GUIDANCE ONLY. IF USED AS PART OF A REVIEW PROCEDURE FOR PUBLICATION, IT SHOULD NOT REPLACE THE EXPERTISE OF AN EXPERIENCED CRYSTALLOGRAPHIC REFEREE.

No syntax errors found. CIF dictionary Interpreting this report

Datablock: lcz045

Bond precision:	C-C = 0.0182 A		Wavelength=1.54184
Cell:	a=8.2311(1)	b=20.4500(2)	c=21.1854(2)
	alpha=90	beta=98.539(1)	gamma=90
Temperature:	100 K		

	Calculated	Reported
Volume	3526.52(6)	3526.52(6)
Space group	P 21/c	P 1 21/c 1
Hall group	-P 2ybc	-P 2ybc
Moiety formula	C15 H3 Ag3 F18 N6, C15 H12 O3	C15 H3 Ag3 F18 N6, C15 H12 O3
Sum formula	C30 H15 Ag3 F18 N6 O3	C30 H15 Ag3 F18 N6 O3
Mr	1173.09	1173.09
Dx,g cm-3	2.210	2.209
Z	4	4
Mu (mm-1)	14.544	14.544
F000	2256.0	2256.0
F000'	2266.73	
h,k,lmax	10,25,26	10,25,26
Nref	7541	7198
Tmin,Tmax	0.076,0.047	0.333,1.000
Tmin'	0.012	

Correction method= # Reported T Limits: Tmin=0.333 Tmax=1.000
AbsCorr = MULTI-SCAN

Data completeness= 0.955 Theta(max)= 77.930

R(reflections)= 0.1155(5894) wR2(reflections)= 0.3048(7198)

S = 1.172 Npar= 491

The following ALERTS were generated. Each ALERT has the format
test-name_ALERT_alert-type_alert-level.
Click on the hyperlinks for more details of the test.

Alert level B

PLAT971_ALERT_2_B Check Calcd Resid. Dens. 0.88Ang From Ag03 3.13 eA-3

Author Response: This Alert is due to presence of residual density in the presence of heavy metal atom (Ag).

PLAT971_ALERT_2_B Check Calcd Resid. Dens. 0.83Ang From Ag03 2.82 eA-3

Author Response: This Alert is due to presence of residual density in the presence of heavy metal atom (Ag).

PLAT971_ALERT_2_B Check Calcd Resid. Dens. 0.96Ang From Ag01 2.67 eA-3

Author Response: This Alert is due to presence of residual density in the presence of heavy metal atom (Ag).

PLAT971_ALERT_2_B Check Calcd Resid. Dens. 0.83Ang From Ag02 2.59 eA-3

Author Response: This Alert is due to presence of residual density in the presence of heavy metal atom (Ag).

PLAT971_ALERT_2_B Check Calcd Resid. Dens. 0.95Ang From Ag01 2.52 eA-3

Author Response: This Alert is due to presence of residual density in the presence of heavy metal atom (Ag).

Alert level C

```
PLAT082_ALERT_2_C High R1 Value .................................       0.12 Report
PLAT084_ALERT_3_C High wR2 Value (i.e. > 0.25) ..................       0.30 Report
PLAT213_ALERT_2_C Atom F00R             has ADP max/min Ratio .....      3.2 prolat
PLAT234_ALERT_4_C Large Hirshfeld Difference F00A     --C01L     .       0.17 Ang.
PLAT309_ALERT_2_C Single Bonded Oxygen (C-O > 1.3 Ang) ..........       O00N Check
PLAT342_ALERT_3_C Low Bond Precision on  C-C Bonds ..............    0.01818 Ang.
```

```
PLAT362_ALERT_2_C Short  C(sp3)-C(sp2) Bond  C015     - C01F     .       1.41 Ang.
PLAT362_ALERT_2_C Short  C(sp3)-C(sp2) Bond  C00Z     - C013     .       1.40 Ang.
PLAT906_ALERT_3_C Large K Value in the Analysis of Variance ......     30.567 Check
PLAT906_ALERT_3_C Large K Value in the Analysis of Variance ......      5.656 Check
PLAT906_ALERT_3_C Large K Value in the Analysis of Variance ......      2.738 Check
PLAT911_ALERT_3_C Missing FCF Refl Between Thmin & STh/L=    0.600         41 Report
                0  0  2,   8  1  5,   5 20  5,   8  1  6,   3 20  7,  -3 22  9,
                8  0 10,   8  1 10,   8  2 10,   3 18 10,   8  1 11,   0 19 13,
                5 12 15,   4  6 16,   4  7 16,   4  8 16,   3  9 16,   3 10 16,
                4 10 16,   4 11 16,   3 12 16,   3 13 16,   3  9 17,   3 13 17,
                4  0 18,   3 13 18,   4  2 19,   4  3 19,   2  7 19,   1  8 19,
                   (   11 More Missing: see the .ckf listing file)
PLAT934_ALERT_3_C Number of (Iobs-Icalc)/Sigma(W) > 10 Outliers ..          1 Check
                2  9 20,
PLAT971_ALERT_2_C Check Calcd Resid. Dens.  1.26Ang From Ag02           2.49 eA-3
```

Author Response: This Alert is due to presence of residual density in the presence of heavy metal atom (Ag).

```
PLAT971_ALERT_2_C Check Calcd Resid. Dens.  0.85Ang From Ag02           2.19 eA-3
```

Author Response: This Alert is due to presence of residual density in the presence of heavy metal atom (Ag).

```
PLAT971_ALERT_2_C Check Calcd Resid. Dens.  1.25Ang From C016           1.96 eA-3
```

Author Response: This Alert is due to presence of residual density in the presence of heavy metal atom (Ag).

```
PLAT971_ALERT_2_C Check Calcd Resid. Dens.  0.92Ang From C00Z           1.95 eA-3
```

Author Response: This Alert is due to presence of residual density in the presence of heavy metal atom (Ag).

```
PLAT971_ALERT_2_C Check Calcd Resid. Dens.  1.26Ang From Ag02           1.93 eA-3
```

Author Response: This Alert is due to presence of residual density in the presence of heavy metal atom (Ag).

```
PLAT971_ALERT_2_C Check Calcd Resid. Dens.  1.28Ang From Ag02           1.79 eA-3
```

Author Response: This Alert is due to presence of residual density in the presence of heavy metal atom (Ag).

```
PLAT971_ALERT_2_C Check Calcd Resid. Dens.  0.56Ang From C011            1.76 eA-3
```

Author Response: This Alert is due to presence of residual density in the presence of heavy metal atom (Ag).

```
PLAT971_ALERT_2_C Check Calcd Resid. Dens.  1.13Ang From F00P            1.63 eA-3
```

Author Response: This Alert is due to presence of residual density in the presence of heavy metal atom (Ag).

```
PLAT971_ALERT_2_C Check Calcd Resid. Dens.  1.96Ang From O00T            1.63 eA-3
```

Author Response: This Alert is due to presence of residual density in the presence of heavy metal atom (Ag).

```
PLAT971_ALERT_2_C Check Calcd Resid. Dens.  0.60Ang From C01H            1.63 eA-3
```

Author Response: This Alert is due to presence of residual density in the presence of heavy metal atom (Ag).

```
PLAT971_ALERT_2_C Check Calcd Resid. Dens.  1.31Ang From Ag03            1.55 eA-3
```

Author Response: This Alert is due to presence of residual density in the presence of heavy metal atom (Ag).

```
PLAT971_ALERT_2_C Check Calcd Resid. Dens.  1.01Ang From F00H            1.52 eA-3
```

Author Response: This Alert is due to presence of residual density in the presence of heavy metal atom (Ag).

```
PLAT971_ALERT_2_C Check Calcd Resid. Dens.  2.25Ang From F00G            1.51 eA-3
```

Author Response: This Alert is due to presence of residual density in the presence of heavy metal atom (Ag).

```
PLAT971_ALERT_2_C Check Calcd Resid. Dens.  1.27Ang From Ag01            1.51 eA-3
```

Author Response: This Alert is due to presence of residual density in the presence of heavy metal atom (Ag).

```
PLAT972_ALERT_2_C Check Calcd Resid. Dens.  2.11Ang From F00G           -1.93 eA-3
PLAT972_ALERT_2_C Check Calcd Resid. Dens.  0.33Ang From C015           -1.92 eA-3
PLAT972_ALERT_2_C Check Calcd Resid. Dens.  0.79Ang From Ag03           -1.87 eA-3
PLAT972_ALERT_2_C Check Calcd Resid. Dens.  1.87Ang From F008           -1.80 eA-3
```

```
PLAT972_ALERT_2_C Check Calcd Resid. Dens.  0.66Ang From O00T          -1.65 eA-3
PLAT972_ALERT_2_C Check Calcd Resid. Dens.  1.62Ang From C00V          -1.61 eA-3
PLAT972_ALERT_2_C Check Calcd Resid. Dens.  0.99Ang From C01B          -1.59 eA-3
PLAT972_ALERT_2_C Check Calcd Resid. Dens.  1.78Ang From Ag01          -1.58 eA-3
PLAT972_ALERT_2_C Check Calcd Resid. Dens.  0.97Ang From Ag01          -1.57 eA-3
PLAT972_ALERT_2_C Check Calcd Resid. Dens.  0.95Ang From Ag01          -1.57 eA-3
PLAT972_ALERT_2_C Check Calcd Resid. Dens.  1.30Ang From F00G          -1.56 eA-3
PLAT972_ALERT_2_C Check Calcd Resid. Dens.  1.54Ang From Ag02          -1.54 eA-3
PLAT972_ALERT_2_C Check Calcd Resid. Dens.  0.42Ang From N00U          -1.52 eA-3
PLAT977_ALERT_2_C Check Negative Difference Density on H01E      .      -0.41 eA-3
PLAT977_ALERT_2_C Check Negative Difference Density on H00T      .      -1.18 eA-3
PLAT977_ALERT_2_C Check Negative Difference Density on H00X      .      -0.45 eA-3
PLAT977_ALERT_2_C Check Negative Difference Density on H01B      .      -0.65 eA-3
```

Alert level G

```
PLAT002_ALERT_2_G Number of Distance or Angle Restraints on AtSite          3 Note
PLAT003_ALERT_2_G Number of Uiso or U(i,j)  Restrained non-H-Atoms         15 Report
PLAT007_ALERT_5_G Number of Unrefined Donor-H Atoms .............           1 Report
              H00T
PLAT083_ALERT_2_G SHELXL Second Parameter in WGHT  Unusually Large     381.40 Why ?
PLAT142_ALERT_4_G s.u. on b - Axis Small or Missing .............    0.00020 Ang.
PLAT143_ALERT_4_G s.u. on c - Axis Small or Missing .............    0.00020 Ang.
PLAT172_ALERT_4_G The CIF-Embedded .res File Contains DFIX Records          2 Report
PLAT177_ALERT_4_G The CIF-Embedded .res File Contains DELU Records          1 Report
PLAT178_ALERT_4_G The CIF-Embedded .res File Contains SIMU Records          1 Report
PLAT186_ALERT_4_G The CIF-Embedded .res File Contains ISOR Records          3 Report
PLAT242_ALERT_2_G Low    'MainMol' Ueq as Compared to Neighbors of       C012 Check
PLAT242_ALERT_2_G Low    'MainMol' Ueq as Compared to Neighbors of       C01A Check
PLAT242_ALERT_2_G Low    'MainMol' Ueq as Compared to Neighbors of       C01F Check
PLAT242_ALERT_2_G Low    'MainMol' Ueq as Compared to Neighbors of       C01J Check
PLAT242_ALERT_2_G Low    'MainMol' Ueq as Compared to Neighbors of       C01L Check
PLAT720_ALERT_4_G Number of Unusual/Non-Standard Labels ..........         75 Note
              Ag01    Ag02    Ag03    F004    F005    F006    F007    F008
              F009    F00A    F00B    F00C    F00D    F00E    F00F    F00G
              F00H    F00I    N00J    C01H    C01E    H01E    C011    N00L
              O00K    F00M    O00N    F00P    F00R    N00S    C01C    C01M
              H01M    C01D    N00Q    O00T    H00T    N00U    C015    C00X
              H00X    C00Z    N00O    C00W    C00Y    H00Y    C014    C017
              C019    H019    C01I    H01I    C018    H018    C010    C012
              C013    C016    H016    C00V    C01K    H01K    C01N    H01N
              C01O    H01O    C01G    H01G    C01A    C01B    H01A    H01B
              C01F    C01J    C01L
PLAT860_ALERT_3_G Number of Least-Squares Restraints ............          92 Note
PLAT883_ALERT_1_G Absent Datum for _atom_sites_solution_primary ..     Please Do !
PLAT912_ALERT_4_G Missing # of FCF Reflections Above STh/L=  0.600        302 Note
PLAT933_ALERT_2_G Number of HKL-OMIT Records in Embedded .res File          9 Note
                1  8 19,   2  6 20,   2  7 19,   2  7 20,   3  7 20,   3 12 16,
                4  6 16,   4  7 16,   4  8 16,
PLAT941_ALERT_3_G Average HKL Measurement Multiplicity ..........         2.5 Low
PLAT969_ALERT_5_G The 'Henn et al.' R-Factor-gap value ..........       4.462 Note
              Predicted wR2: Based on SigI**2  6.83 or SHELX Weight 26.00
PLAT978_ALERT_2_G Number C-C Bonds with Positive Residual Density.          0 Info
```

---

0 **ALERT level A** = Most likely a serious problem - resolve or explain

5 **ALERT level B** = A potentially serious problem, consider carefully

```
 44 ALERT level C = Check. Ensure it is not caused by an omission or oversight
 23 ALERT level G = General information/check it is not something unexpected

  1 ALERT type 1 CIF construction/syntax error, inconsistent or missing data
 51 ALERT type 2 Indicator that the structure model may be wrong or deficient
  9 ALERT type 3 Indicator that the structure quality may be low
  9 ALERT type 4 Improvement, methodology, query or suggestion
  2 ALERT type 5 Informative message, check
```

Validation response form

Please find below a validation response form (VRF) that can be filled in and pasted into your CIF.

```
# start Validation Reply Form
_vrf_PLAT082_lcz045
;
PROBLEM: High R1 Value .................................       0.12 Report
RESPONSE: ...
;
_vrf_PLAT084_lcz045
;
PROBLEM: High wR2 Value (i.e. > 0.25) ..................       0.30 Report
RESPONSE: ...
;
_vrf_PLAT213_lcz045
;
PROBLEM: Atom F00R            has ADP max/min Ratio .....        3.2 prolat
RESPONSE: ...
;
_vrf_PLAT234_lcz045
;
PROBLEM: Large Hirshfeld Difference F00A     --C01L     .       0.17 Ang.
RESPONSE: ...
;
_vrf_PLAT309_lcz045
;
PROBLEM: Single Bonded Oxygen (C-O > 1.3 Ang) ..........       O00N Check
RESPONSE: ...
;
_vrf_PLAT342_lcz045
;
PROBLEM: Low Bond Precision on  C-C Bonds ..............    0.01818 Ang.
RESPONSE: ...
;
_vrf_PLAT362_lcz045
;
PROBLEM: Short  C(sp3)-C(sp2) Bond  C015     - C01F     .       1.41 Ang.
RESPONSE: ...
;
_vrf_PLAT906_lcz045
;
PROBLEM: Large K Value in the Analysis of Variance ......     30.567 Check
RESPONSE: ...
;
_vrf_PLAT911_lcz045
```

```
;
PROBLEM: Missing FCF Refl Between Thmin & STh/L=    0.600          41 Report
RESPONSE: ...
;
_vrf_PLAT934_lcz045
;
PROBLEM: Number of (Iobs-Icalc)/Sigma(W) > 10 Outliers ..           1 Check
RESPONSE: ...
;
_vrf_PLAT972_lcz045
;
PROBLEM: Check Calcd Resid. Dens.  2.11Ang From F00G           -1.93 eA-3
RESPONSE: ...
;
_vrf_PLAT977_lcz045
;
PROBLEM: Check Negative Difference Density on H01E      .      -0.41 eA-3
RESPONSE: ...
;
# end Validation Reply Form
```

It is advisable to attempt to resolve as many as possible of the alerts in all categories. Often the minor alerts point to easily fixed oversights, errors and omissions in your CIF or refinement strategy, so attention to these fine details can be worthwhile. It is up to the individual to critically assess their own results and, if necessary, seek expert advice.

PLATON version of 17/09/2025; check.def file version of 17/09/2025

---

duplicate check

No duplication found

Datablock lcz045 - ellipsoid plot

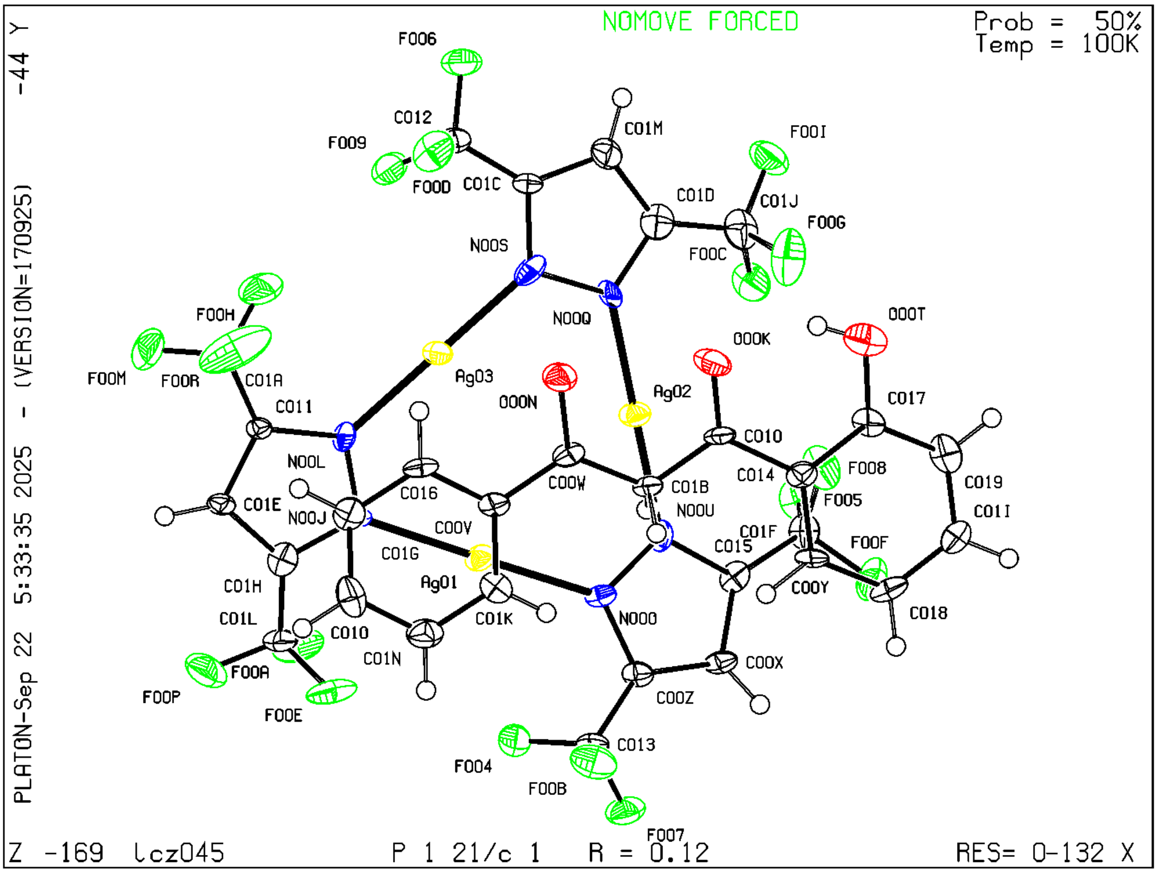

Supplement: Supplementary file 2 — Supporting File 2: anie72462‐sup‐0002‐DataFile.zip. [file ANIE-65-e1218503-s002.zip › supplementary_materials_crystallographic_data submit.zip!/cif_and_checkcif(1-114)/Ag3Pz3_45/Ag3Pz3_45_checkcif.pdf]
